# Enabling Ultrasensitive Photo-detection Through Control of Interface Properties in Molybdenum Disulfide Atomic Layers

**DOI:** 10.1038/srep39465

**Published:** 2016-12-20

**Authors:** Sina Najmaei, Sidong Lei, Robert A. Burke, Barbara M. Nichols, Antony George, Pulickel M. Ajayan, Aaron D. Franklin, Jun Lou, Madan Dubey

**Affiliations:** 1 United States Army Research Laboratories, Sensors and Electron Devices Directorate, 2800 Powder Mill Road, Adelphi, Maryland 20783, USA; 2 Department of Materials Science and NanoEngineering, Rice University, Houston, Texas 77005, USA; 3 Department of Electrical & Computer Engineering, Duke University, Durham, North Carolina 27708, USA

## Abstract

The interfaces in devices made of two-dimensional materials such as MoS_2_ can effectively control their optoelectronic performance. However, the extent and nature of these deterministic interactions are not fully understood. Here, we investigate the role of substrate interfaces on the photodetector properties of MoS_2_ devices by studying its photocurrent properties on both SiO_2_ and self-assembled monolayer-modified substrates. Results indicate that while the photoresponsivity of the devices can be enhanced through control of device interfaces, response times are moderately compromised. We attribute this trade-off to the changes in the electrical contact resistance at the device metal-semiconductor interface. We demonstrate that the formation of charge carrier traps at the interface can dominate the device photoresponse properties. The capture and emission rates of deeply trapped charge carriers in the substrate-semiconductor-metal regions are strongly influenced by exposure to light and can dynamically dope the contact regions and thus perturb the photodetector properties. As a result, interface-modified photodetectors have significantly lower dark-currents and higher on-currents. Through appropriate interfacial design, a record high device responsivity of 4.5 × 10^3^ A/W at 7 V is achieved, indicative of the large signal gain in the devices and exemplifying an important design strategy that enables highly responsive two-dimensional photodetectors.

The study of two-dimensional (2D) materials, covering a wide spectrum of physics and applications, has inspired a great deal of attention. The broad range of bandgap properties^1^, strong light-matter interactions^2^,^3^,^4^, significant spin-orbit entanglement^5^,^6^,^7^,^8^, suitable electronic transport properties^9^, and effective plasmonic interactions^10^,^11^,^12^,^13^,^14^ are several heavily explored research areas that motivate 2D optoelectronic applications. But a comprehensive understanding of the 2D material device properties and light-matter interactions are essential to any progress in this field. Several studies have examined the photodetector and phototransistor properties of devices made using transition metal dichalcogenides (TMDs)^4^,^15^. These studies show promising photodetection efficiency and responsivities. However, a complete understanding of the photocurrent generation and device properties that incorporate the important role of interfaces remains elusive.

The large surface area and lack of dangling bonds in 2D materials make their interfaces with 3D materials in device platforms unique and less understood^16^,^17^,^18^. These interfaces include the interface of the 2D materials with their substrate and the interface of the 2D materials and metal contacts. Substrate interface interactions can result in interfacial charge carrier scattering and carrier mobility changes in 2D materials^19^,^20^,^21^,^22^. They also can result in strong exciton localizations and affect the photoluminescence response^23^. Furthermore, interfacial design can be used to control and modify the 2D device properties^16^. It is known that charge carrier traps in 3D nanomaterials can be used as an important tool for control of photodetector properties^24^,^25^,^26^. For instance ultrasensitive photodetectors made of quantum dots with deliberately designed surface trap functionalization have been demonstrated in photodetectors made of low dimensional material systems^24^. The dynamic behavior of contact interface traps and the nature of charge carrier capture and escape can modify the contact barrier and potential levels in the device and alter its properties. Therefore, the design of traps and controlling their properties has been widely proposed as a unique approach in tailoring the properties of nanomaterial based photodetectors^24^,^25^,^26^. Since 2D materials have large surface to volume ratios, interface properties should have high importance in their optoelectronics.

In this work, we examine the role of interfaces on the photodetector properties of single-layered molybdenum disulfide (MoS_2_). We control the interface properties of the substrate by controlling the interfacial chemistry. Comparing the photodetector properties of MoS_2_ devices on SiO_2_ versus thiol-modified substrates, we demonstrate that interfaces have an important role in controlling the responsivity and response time of the 2D photodetectors. Our results indicate that while the photosensitivity of the photodetectors can be enhanced through interface engineering, the device response time is slightly compromised. We attribute this trade-off to changes in the electrical contact barrier at the metal-semiconductor junction. We conclude that interface modification results in deep interfacial traps at the vicinity of metal contacts, the photo-induced doping effects of which dramatically reduce the contact resistance and result in a major gain in photodetection. However, the resultant dynamic of capture and emission rates lead to low device response times. These results explain a fundamentally important mechanism occurring in the contact regions of 2D photodetectors and establish guidelines for the design of highly sensitive 2D optoelectronics.

## Results

**Figure 1:**
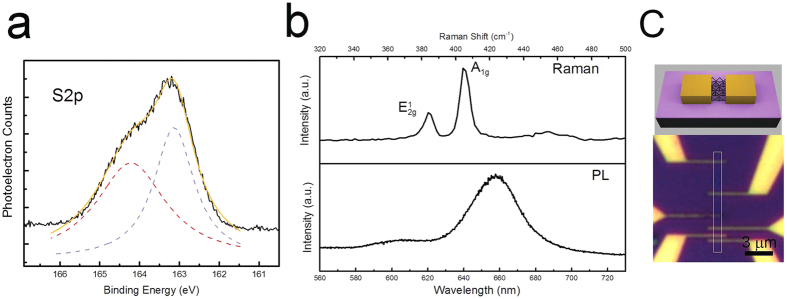
Basic characterization and device designs for measurements. (**a**) S2p doublet peak from the SAMs modified surfaces obtained using XPS Al K_α_ x-ray radiation. (**b**) The Raman and PL spectra acquired from CVD MoS_2_ on SiO_2_/Si substrates used in this study. (**c**) Device geometries used for electronic and optoelectronic characterization. The width of the devices varies between 1–3 μm and the channel lengths vary between 0.2–3 μm.

The control of interfaces is a strong tool for modifying the properties of devices based on 2D materials^16^. One straightforward approach for modification of interfaces is the application of self-assembled monolayers (SAMs). The diversity in the chemistry, MOSFET compatibility, and functionality of SAMs are characteristics that make them an attractive choice for such applications. Thiol-based organosilanes are an extensively studied and diverse class of SAMs that show great promise in the modification of SiO_2_ interfaces^27^. In this study, we use thiol-terminated (3-Mercaptopropyl) methyldimethoxysilane (CH_3_Si(OCH_3_)_2_CH_2_CH_2_CH_2_SH) to modify the interface chemistry of SiO_2_ substrates. To prepare the SAMs we first treat the SiO_2_ substrates with oxygen plasma for 5 minutes to remove all organic residues and promote the formation of dense surface hydroxyl groups. Next, we place the substrates and a drop of organosilane precursor in a vacuum desiccator under moderate vacuum conditions (~5 mBar at 60 °C) for roughly 2 hours. The prepared -SH/SiO_2_ has a significantly larger contact angle (~65 degrees) as compared to pristine SiO_2_ (~40 degrees), representative of the enhanced hydrophobicity of the SAM-modified surface. The x-ray photoelectron spectra (XPS) acquired from the as-grown SAMs show a doublet peak, S2p_3/2_ at 163 eV and S2p_1/2_ at 164 eV, associated with the unbound S2p sulfur bonds in the thiolate chemistry and further support our observation of interface modifications (Fig. 1a). MoS_2_ samples were prepared using a common CVD recipe and transferred to the -SH/SiO_2_ substrates as well as to baseline, pristine 300 nm thick thermally oxidized P-type Si substrates^28^. The transfer process consists of coating the CVD-grown MoS_2_ with PMMA and releasing it from the substrate in KoH solution. The PMMA/MoS_2_ samples are transferred to the desired substrates and the PMMA film is removed using acetone solution. We will refer to the SAMs modified interface as MoS_2_-SH/SiO_2_ and use the MoS_2_-SiO_2_ to describe bare SiO_2_ interface. The samples were then characterized using Raman and photoluminescence (PL) spectroscopy. The spacing between the E^1^_2g_ and A_1g_ Raman peaks, ~20 cm^−1^, and the intense PL spectrum are indicative of the high quality and monolayer nature of the MoS_2_ samples (Fig. 1b). We use e-beam lithography and oxygen plasma etch to pattern source/drain metal contacts and channel MoS_2_ geometry to yield bottom-gated transistors on both the baseline and SAM-modified substrates. We use e-beam evaporation at ~5 × 10^−6^ Torr to deposit 3 nm/50 nm thick Ti/Au metals as contact metal. The devices include single- and multi-channel length designs suitable for typical field-effect characterization, optoelectronic measurements, and contact resistance estimation using the transmission line method (TLM) (Fig. 1c).

**Figure 2:**
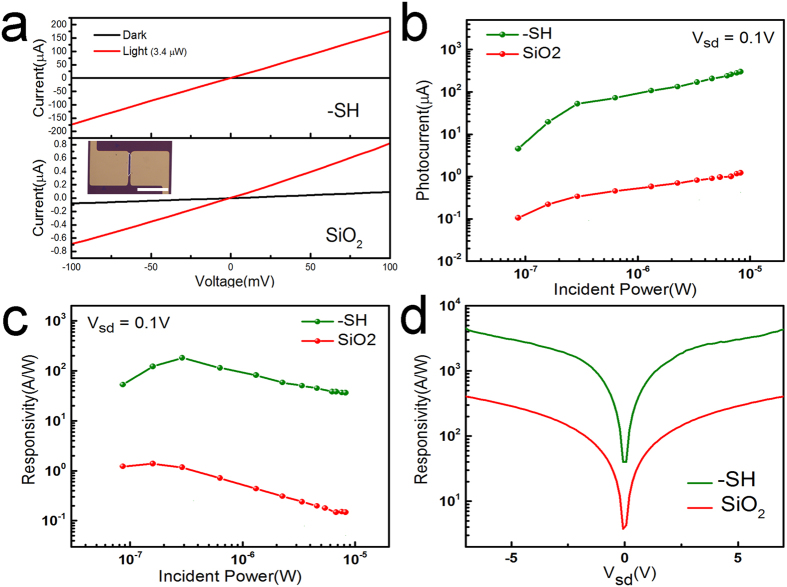
Photocurrent and photoresponse properties of interface-controlled MoS_2_ devices. (**a**) Current-voltage experiments under dark and light conditions for devices with MoS_2_-SH/SiO_2_ and MoS_2_-SiO_2_ interfaces. Inset shows a typical device, 40 μm wide with 3 μm long channels with large contact pads, used in the photocurrent experiments. The scale bar is 30 μm in the inset. (**b**) Incident power dependence of photocurrent, (**c**) responsivity and its incident power dependence, and bias voltage dependency of responsivity in devices with both types of interfaces.

To perform optoelectronic measurements on our samples, we use a 532 nm laser with a beam diameter of 150 μm. The beam is centered in such a way that the entire active device, including the 2D material, is in the incident light region. The laser power is adjusted using an acoustic modulator and perform photocurrent measurements. First, the current-voltage characteristics are measured for devices (40 μm wide with 3 μm channel lengths) in dark and illuminated conditions with an emphasis on understanding the influence of interface changes. An incident laser power of 3.4 μW was used for these experiments. Results show a clear distinction between the behavior of devices made on SiO_2_ and -SH/SiO_2_. A major increase in the conductance of the device, roughly by 4 orders of magnitude, is indicative of a surprisingly high device response in MoS_2_-SH/SiO_2_ samples (Fig. 2a). These results, as compared to only one order of magnitude change in conductance for similar devices on SiO_2_, exemplify a major difference in optical detection properties brought about by the SAM surface modification. In these experiments, the dark current in MoS_2_-SH/SiO_2_ devices measured at 0.1 V was close to one order of magnitude smaller than MoS_2_-SiO_2_ devices. Meanwhile, when illuminated, the current in MoS_2_-SH/SiO_2_ devices increased to roughly two orders of magnitude larger than that of MoS_2_-SiO_2_ devices. The lower dark current, as well as the large photocurrent generation in MoS_2_-SH/SiO_2_ devices, contributes significantly to the differences seen in the device characteristics.

To future explore the significant role of interface traps on the photodetector characteristics of these devices, the photoresponse and light power dependency of the photocurrent were measured (Fig. 2b). At lower power, the photocurrent rapidly increases in both MoS_2_-SiO_2_ and MoS_2_-SH/SiO_2_ interface conditions, with the changes leveling out at higher powers. The photocurrent is steadily close to two orders of magnitude larger in MoS_2_-SH/SiO_2_ interface conditions as compared to MoS_2_-SiO_2_. The device responsivity for varied interfaces is calculated to further compare the performance from these two interface conditions. The responsivity at wavelength (*λ*), 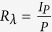 (*I*_*P*_ being the photocurrent and *P* the incident light power) is a measure of the photodetector’s effectiveness in converting light power to electrical current. The responsivity in these devices sharply increases to a maximum and then gradually decreases as the incident power is ramped up (Fig. 2c). Owing to the direct proportionality to the photocurrent, the photoresponse in MoS_2_-SH/SiO_2_ devices also exhibits an increase of two orders of magnitude over that of MoS_2_-SiO_2_ devices. The photocurrent and, as a result, responsivity of the device is also highly dependent on the applied bias voltage. We decipher these effects by measuring the responsivity as a function V_ds_ up to 7 V (Fig. 2d). The measurements for MoS_2_-SH/SiO_2_ samples show record high values for responsivity in MoS_2_ devices, with at least one order of magnitude enhancement. External quantum efficiency is related to the device responsivity through 
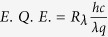
, where *h* is the plank constant, *c* is the speed of light, and *q* is the electron charge. The external quantum efficiencies in our devices reach maximum values close to 1000% and 10000%, for MoS_2_-SiO_2_ and MoS_2_-SH/SiO_2_ devices, respectively. Since the absorption in 2D materials is small, the extraordinary interface-dependent gain in these devices that result in their large responsivities and quantum efficiencies needs to be explained.

**Figure 3:**
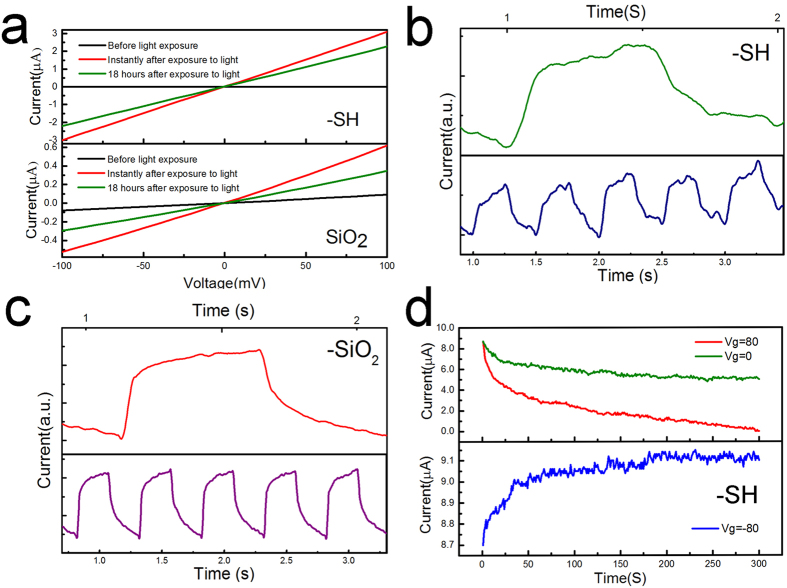
Photocurrent response. (**a**) Current-voltage experiments before and after exposure to light in MoS_2_-SH/SiO_2_ and MoS_2_-SiO_2_ samples. Time resolved photocurrents recorded at 3.4 μW light power, 2 Hz modulation frequency, and V_ds_ = 1 V in (**b**) MoS_2_-SH/SiO_2_ & (**c**) MoS_2_-SiO_2_ samples. (**d**) The changes in current for dark characteristics of MoS_2_-SH/SiO_2_ as a function of time and gate voltage.

We further examine the temporal properties of our detector devices to improve understanding of the response time properties. A distinct feature of the current-voltage behavior in the devices was the consistent dependence on history of exposure to light; the devices with many hours exposure to light show higher conductance even if they were left in dark environments. The slow recovery of the device conductance to values before exposure to light suggests that a slow dynamic process is involved. We measure the dark current-voltage characteristics of the devices before and after 30 min of exposure to 3.4 μW light, as shown in Fig. 3a. The conductivity increases by several orders of magnitude and remains high for a period in excess of 18 hours. These effects are more prominent in the MoS_2_-SH/SiO_2_ samples. Time resolved photocurrent measurements were also performed to examine the photoresponse of the devices at a modulation frequency of 2 Hz. In Fig. 3b,c, five cycles of the measurements are shown as well as a close up of one representative cycle for further analysis. As is evident, there is a gradual increase in the average signal measurements in MoS_2_-SH/SiO_2_, and the measurements are slightly noisy. However, this gradual increase in current is not as dramatic in the MoS_2_-SiO_2_ devices. The distinct interface dependent behavior of rise and fall times in the device signal resembles what we have observed in Fig. 3a. The MoS_2_-SH/SiO_2_ devices show slower rise and fall times as compared to MoS_2_-SiO_2_ devices. In addition, the long tail in both rise and fall characteristics suggest the involvement of two mechanisms of signal generation that needs to be determined. The net effect of these two mechanisms results in rise times of 140 ms and 96 ms for MoS_2_-SH/SiO_2_ and MoS_2_-SiO_2_, respectively. The fall times for these conditions amount to 186 ms and 151 ms for MoS_2_-SH and MoS_2_-SiO_2_, respectively.

Next, we focus on investigating the dynamic behavior of the signal in a MoS_2_-SH/SiO_2_ sample by measuring the time dependency of current. After exposing the devices to 30 min of 3.4 μW light the current changes were measured over time at varied gate voltages in dark conditions (Fig. 3d). In this experiment, a familiar behavior is observed where initially the signal drops quickly, followed by a gradual and lengthy tail with zero and positive gate voltages. This behavior leads to a long recovery time of the device to its original state as depicted in Fig. 3a. The measurements for the zero and positive 80 V gate voltages are shown in the top panel of Fig. 3d. The measured current has been manually shifted, by subtracting a constant value from the positively gated signal, to match the starting currents in the two devices and allow for a better comparison between the different voltage conditions. As a result the magnitude of the signal is arbitrary, but the changes represent the distinct time-dependent behavior of the current and its relation to gate voltage.

The faster decay of the signal in the positive vs zero gate voltage conditions and the continuous increase in the signal under the negatively gated conditions (in this case −80 V), presents a significantly contrasting behavior. As the decay and recombination of excitonic pairs in a free excitonic system are in the picosecond time scales, a different mechanism with long life times should be responsible for the observed behavior. The trapping of the carriers at the interface and its resultant modification of device contact resistance is a common observation in nanomaterial-based photodetectors that leads to increased gain and photoresponsivity^24^,^25^,^26^. Formation of such donor type interface electron traps at the junction and the vicinity of the metal-semiconductor-SAMs are postulated to be the primary source of interaction that govern the photodetector properties in the present devices. Exposing the contact regions to light will excite the trapped electrons in these sites and dope the material, which will amount to significant changes in the contact barrier properties. To explain this more clearly we will now focus on the contact resistance properties of the devices.

**Figure 4:**
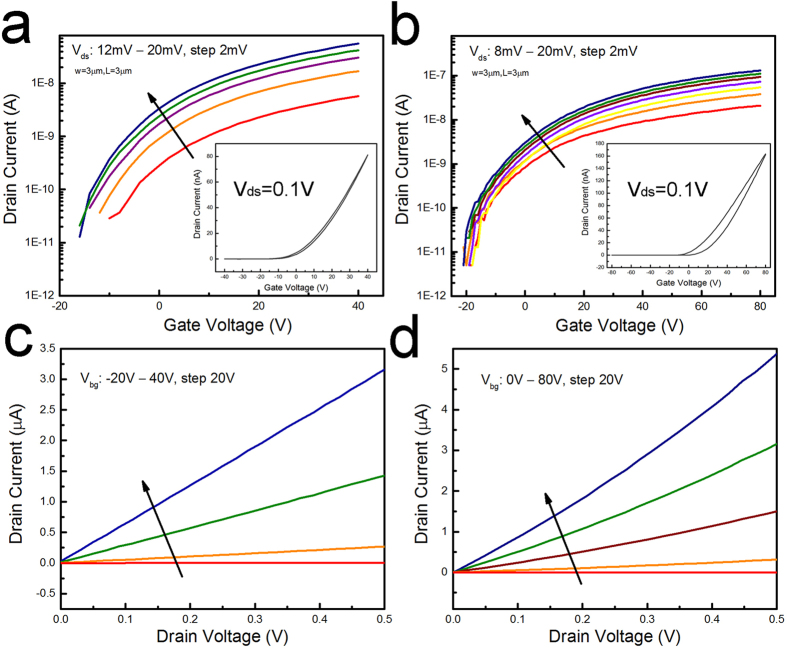
Back gated field-effect behavior of MoS_2_-SiO_2_ and MoS_2_-SH/SiO_2_ devices. The logarithmic scale transfer curves for (**a**) MoS_2_-SiO_2_ devices while V_ds_ is changed between 12 mV and 20 mV & (**b**) MoS_2_-SH/SiO_2_ devices while V_ds_ is changed between 8 mV and 20 mV. Insets represent the linear scale transfer curves for these devices demonstrating the on-current and hysteresis. The output curves for (**c**) MoS_2_-SiO_2_ devices while V_bg_ is changed between −20 and 40 V & (**d**) MoS_2_-SH/SiO_2_ devices with V_bg_ changed between 0 and 80 V.

To understand these results and the origin of changes in the device properties we look at the general electronic transport and device properties of MoS_2_ on SiO_2_ and -SH/SiO_2_ surfaces and assess some of the main distinctions resulting from the interface alterations. The transfer curves acquired for varied drain-source voltages are shown in Fig. 4a,b for MoS_2_-SiO_2_ and MoS_2_-SH/SiO_2_, respectively. A clear change in threshold voltage, subthreshold behavior, and hysteresis of the devices are observable. The threshold voltages estimated from the transfer curves presented in the inset of Fig. 4a,b at V_ds_ = 0.1 V are −1 V and 11 V for MoS_2_ on SiO_2_ and on -SH/SiO_2,_ respectively. The subthreshold swings estimated for these devices also vary from 6 V/decade in MoS_2_-SiO_2_ to 4 V/decade in MoS_2_-SH/SiO_2_. The relatively high subthreshold swings are attributed to the limited gate control in these devices based on the 300 nm SiO_2_ bottom gates. Even still, the lower subthreshold swing values in -SH/SiO_2_ supported devices is indicative of the passivation role of the SAM for reducing interface trap charge states and the significant modification of band tails that extend into the MoS_2_ band gap^22^. The trap states at the substrate interface, resulting from point defects and 2D interfaces, can degrade both the mobility and switching characteristics of the device^19^,^22^. The hysteresis is significantly larger for the MoS_2_-SH/SiO_2_ samples, at 23.7 V compared to the MoS_2_-SiO_2_ with 2.5 V. We suspect that a field-induced change in the interface states of the metal-semiconductor is to blame for the observed hysteresis; however, further examination of this behavior is needed for a more definitive answer.

The output curves shown in Fig. 4c,d further distinguish the impact of the SAM-modified interface on MoS_2_ device performance. The zero back gate voltage sheet resistances are roughly two orders of magnitude larger in MoS_2_-SH/SiO_2_ samples. The sheet resistance of a MoS_2_-SiO_2_ device is 2 M Ω/◽ while the MoS_2_-SH/SiO_2_ sample has a sheet resistance of 371 MΩ/◽. The slight nonlinearity in the IV characteristics of the MoS_2_-SH/SiO_2_ samples at low fields compared to MoS_2_-SiO_2_ samples suggests a difference in the electron transport at the metal-semiconductor junctions. From these experiments we conclude that the observed interface-dependent differences in the device properties are highly related to the role of contact barrier properties.

**Figure 5:**
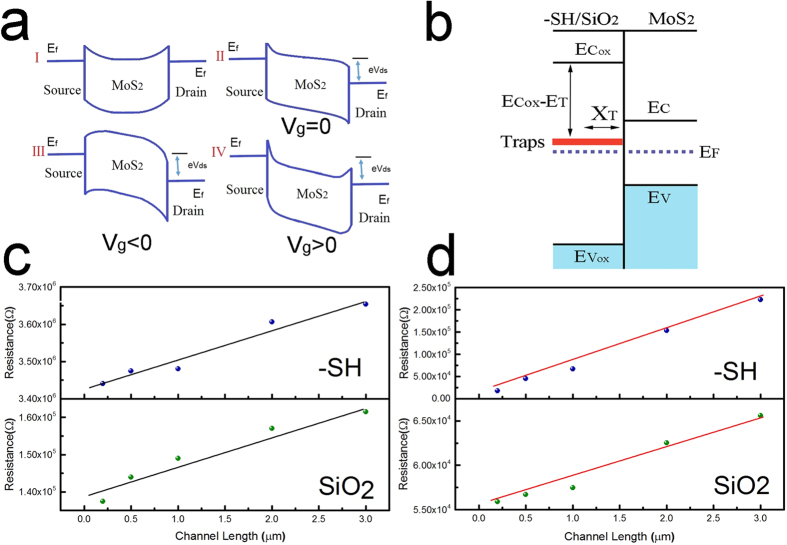
Contact resistance of MoS_2_ and the role of interface states. (**a**) The band diagram for MoS_2_ and formation and gate voltage dependence of contact barriers at the metal semiconductor junction. (**b**) Energy band diagram of MoS_2_-SH/SiO_2_ and the representation of deep trap states at the substrate interface. Transfer length measurements for MoS_2_ samples on varied interfaces (**c**) before light exposure and (**d**) after light exposure.

Energy band diagrams of the devices highlighting four relevant conditions at the metal-semiconductor contact are given in Fig. 5a. At the contact, the Fermi energy level of the metal (Au) is lower than the bottom of MoS_2_ conduction band energy which results in bending of the bands and an electric field that depletes regions of the material of free electrons. This forms the contact barrier shown in Part I. If we apply a bias across the device, the electric field will slightly modify the barrier (Part II). By applying a gate voltage, the barrier width will change as shown in Parts III & IV. Therefore, to explain the role of traps on the device contact resistance, we need to consider the doping effects and the dynamic nature of trap capture and emission^29^. It is known that the doping of contact regions will result in contact resistance changes that follow the relationship (1):


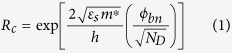


where *N*_*D*_ is the doping density, *ε*_*s*_ is the relative semiconductor (MoS_2_) permittivity, m^*^ is the effective carrier mass, *h* is the Plank constant, and *ϕ*_*bn*_ is the barrier potential. In our photodetector devices, emission of the deeply trapped electrons is promoted by the illuminated condition. This would result in a significant reduction of the contact resistance due to a trap induced increase in the doping density. The process of electron capture and emission after exposure to light and the transient doping of the contact barrier control the time dependent device properties. Electrons are captured and emitted from the trap sites using a phonon assisted process and depend on the capture and emission activation energies. The capture and emission rates, 1/*τ*_*c*_ and 1/*τ*_*e*_, are related to several important trap, oxide gate, and semiconductor properties and can be expressed using the following relationship:


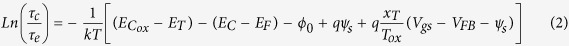


where *τ*_*c*_ and *τ*_*e*_ are the capture and emissions times, 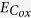 is the conduction band edge of the oxide (-SH/SiO_2_), *E*_*T*_ is the trap energy, *E*_*C*_ is the conduction band edge of MoS_2_, *E*_*F*_ is the Fermi energy, *ϕ*_0_ is the difference between the electron affinities of MoS_2_ and -SH/SiO_2_, *ψ*_*s*_ is the amount of band bending, *x*_*T*_ is the position of the trap measured from the MoS_2_-SH/SiO2 interface, *T*_*ox*_ is the oxide thickness, *V*_*gs*_ is the gate source voltage, and *V*_*FB*_ is the flat band voltage. This is better visualized in the energy band diagram depicted in Fig. 5b. This relationship describes the application of a positive or negative gate voltage that would have opposite effects on the trapping time properties and contact barrier. It is known that *τ*_*e*_ is weakly dependent on the gate voltage^29^. A positive gate voltage would therefore decrease the capture time while a negative gate voltage will increase it, having a direct effect on the dynamic nature of device contact resistance. In dark conditions, the traps will capture electrons and enforce a low doping level regime at the junction. Comparing to the low trap density of MoS_2_-SiO_2_ devices, this will result in higher contact resistance and explains the lower conductance in MoS_2_-SH/SiO_2_ devices (Fig. 3a). Exposing the samples to light excites the electrons into the conduction band and dopes the contact regions, lowering the contact resistance. This explains the significant photocurrent generation in MoS_2_-SH/SiO_2_ devices (Fig. 3a). In transition between the dark and light conditions, the contact resistance and device properties are subject to the competing carrier capture and emission and the diffusion of the trap sites into and out of the contact barrier regions. In zero gate voltage conditions, exposure to light will gradually excite the traps. The planar electric fields will also gradually diffuse more traps into the contact barrier region. In dark conditions, the traps will slowly capture the free carriers and result in a gradual increase in the contact resistance (Fig. 4d top panel). If we apply a positive gate voltage, the trapping capture times, *τ*_*c*_, will decrease and the electron capture rates will increase. The net effect will result in a rapid increase in the contact resistance and a drop in the device signal (Fig. 4d top panel). With an applied negative gate voltage, the *τ*_*c*_ increases and the capture rate decreases. These effects in addition to the field induced diffusion of traps contribute to the contact barrier changes. Migration of defects in 2D materials has previously been used to explain memristor device characteristics in MoS_2_ devices with varied concentrations of sulfur vacancies^30^,^31^. These results suggest that point defects can migrate in these materials from high density defect regions, such as grain boundaries, in large enough electric fields (~1 MV/m). The field assisted diffusion of the traps into the contact regions contribute to the initial device contact resistance decrease. Over time the diffusion of traps into the contact regions is balanced by a decrease in the electron capture and the contact resistance and device current reach an equilibrium state (Fig. 4d bottom panel).

To directly confirm these propositions and comprehend the role of metal-semiconductor-oxide interfaces in our device, we assess the contact resistances in our devices using Transfer Length Measurements (TLM) (Fig. 5c). According to these measurements, the contact resistance before light exposure for MoS_2_-SH/SiO_2_ and MoS_2_-SiO_2_ is 3400 kΩ·μm and 140 kΩ·μm, respectively. Immediately after exposure to light, the contact resistances measured for the same devices changes to 3 kΩ·μm and 25 kΩ·μm for MoS_2_-SH/SiO_2_ and MoS_2_-SiO_2_ devices, respectively (Fig. 5d). This indicates that the contact resistance for both interface conditions is dependent on the exposure to light. This is especially true for the MoS_2_-SH/SiO_2_ devices where the contact resistance is found to decrease by three orders of magnitude, which would explain the significant increase in photosensitivity of the devices. We can also compare the sheet resistances of the channel material (slope of the TLM fit) in devices made on different interfaces before and after light exposure. The sheet resistances for MoS_2_-SH/SiO_2_ and MoS_2_-SiO_2_ devices before exposure to light are 80 KΩ/◽ and 8 KΩ/◽, respectively. After exposure to light, the sheet resistance decreased slightly to 73 KΩ/◽ and 6 KΩ/◽ for MoS_2_-SH/SiO_2_ and MoS_2_-SiO_2_ samples, respectively. The difference between the sheet resistance for each interface can be attributed, in addition to expected sample to sample changes, to the doping levels resulting from charge transfer from the 2D material to the SAMs^16^. The small changes to the sheet resistance of the devices before and after exposure to light are an additional confirmation of the importance of contact resistance to the behavior of our devices. Our results directly link the photocurrent characteristics of MoS_2_-SH/SiO_2_ devices to their dynamic contact resistance properties. It is evident that the greater part of the gain in the photocurrent of the devices is a result of the higher contact resistance change in MoS_2_-SH/SiO_2_ devices. The low dark currents in MoS_2_-SH/SiO_2_ devices combined with significantly higher photocurrent due to doping induced changes in the device contact resistance results in the significant photoresponse in these devices.

**Figure 6:**
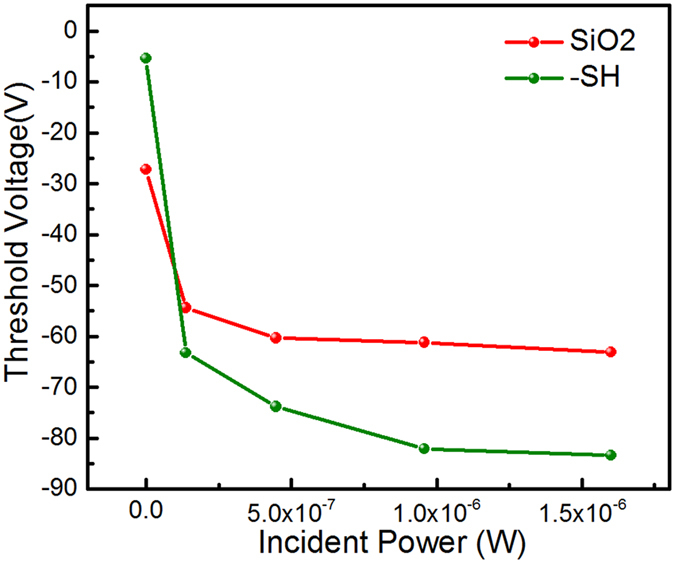
The changes in the threshold voltage acquired from the transfer curves from devices made on MoS_2_-SiO_2_ and MoS_2_-SH/SiO_2_ interfaces.

The proposed mechanisms also explain the behavior of responsivity as the incident power increases. The general behavior of the photo-detector is markedly influenced by the magnitude of the interface trap population near the contact regions. The population of excited trap states are closely related to the trapping and de-trapping time constants as well as the incident light intensity. As the intensity of light increases the population of excited traps increases. The excited trap population reaches an equilibrium at higher intensities where most traps have been excited. This results in a gradual decrease in the responsivity of the photodetectors as the contact resistance changes slow down because of the saturation in doping level. We examine the trap characteristics by measuring the transfer curve threshold voltages, acquired from the devices after every measurement at different incident power (Fig. 6). It is clear that the magnitude of threshold voltage changes is significantly larger in the MoS_2_-SH/SiO_2_ as compared to MoS_2_-SiO_2_ devices. The changes in the threshold voltage of the devices relative to the dark current devices can be attributed to doping. Since the sheet resistance of the channel material does not change much after exposure to light (Fig. 5) we can attribute the threshold voltage changes to increase in contact region doping. The magnitude of doping can be estimated by measuring the changes in threshold voltage (*n*_*T*_ = *ε*_*o*_*ε*Δ*V*_*T*_/*te*, where *ε*_*o*_ and *ε* are the permittivities of free space and SiO_2_, respectively; e is the electron charge; t is the thickness SiO_2_ layer (285 nm); and Δ*V*_*T*_ is the threshold voltage changes). The Δ*V*_*T*_ estimated in the dark and light condition at incident powers close to saturation of threshold voltages, can be used to roughly estimate the trap population. The estimated number of traps in MoS_2_-SH/SiO_2_ and MoS_2_-SiO_2_ interfaces is 6.1 × 10^16^ and 2.8 × 10^16^, respectively. We conclude that the higher number of traps in MoS_2_-SH/SiO_2_, by roughly a factor of 2, enables the markedly high responsivity in the photodetectors made on this interface.

We demonstrate that self-assembled monolayers are a powerful tool for control of oxide interfaces and can be used to modify the interface properties of 2D MoS_2_ devices. The influence of interface interactions were found to be related to the defect energy states they generate in the bandgap of a 2D material. The shallow and deep states lead to trapping of the free carriers and affect a variety of device properties. Our results demonstrate that substrate interface properties have a direct role in the photodetector properties of 2D MoS_2_ devices. We show that the excitation and trapping of carriers at the interface in the affinity of the metal-semiconductor contact regions can dope and modify its barrier properties. These effects slowly change the contact resistance of the devices after exposure to light. The net effect of the interactions results in extremely high device responsivities but also somewhat compromises their response speeds. We conclude that the control of trap properties at the interfaces of 2D materials is a highly effective design strategy and a substantial tool for control of their device properties.

## Methods

The photo-detection characterization was performed using a controlled environment home-built probe station with a 1 inch fused silica optical window for sample illumination and vacuum capability of 10–6 Torr. A 543 nm wavelength He/Ne laser was used as the lights source. An acoustic optical modulator (AOM) was used for laser intensity stabilization, control, and modulation (for light pulse generation). The turn-on and turn-off time constants of the couple laser-AOM is measured to be less than 150 ns. After passing through AOM, laser was directed to the optical window and focused onto the sample placed inside the vacuum probe station. The devices were powered with a Keithley 2634B dual-channel source meter unit connected to the probe station with a triaxial cable for low-noise measurement. The read-out currents were amplified by a Standford Research Systems (SR570) low-noise current amplifier and recorded by a 10 MHz bandwidth Teletronix oscilloscope.

## Additional Information

**How to cite this article**: Najmaei, S. *et al*. Enabling Ultrasensitive Photo-detection Through Control of Interface Properties in Molybdenum Disulfide Atomic Layers. *Sci. Rep.* **6**, 39465; doi: 10.1038/srep39465 (2016).

**Publisher's note:** Springer Nature remains neutral with regard to jurisdictional claims in published maps and institutional affiliations.
